# Smoking Cessation at the Community Pharmacy: Determinants of Success from a Real-Life Practice

**DOI:** 10.3390/pharmacy9030143

**Published:** 2021-08-19

**Authors:** Mónica Condinho, Isabel Ramalhinho, Carlos Sinogas

**Affiliations:** 1 AcF—Acompanhamento Farmacoterapêutico Lda, 7490-324 Pavia, Portugal; monica.condinho@ac-ft.pt (M.C.); sinogas@uevora.pt (C.S.); 2 Faculdade de Ciências e Tecnologia, Universidade do Algarve, 8005-139 Faro, Portugal; 3 Escola de Saúde e Desenvolvimento Humano, Universidade de Évora, 7000-671 Évora, Portugal

**Keywords:** smoking cessation, determinants, community pharmacy, pharmacist, Portugal

## Abstract

The objectives of this study are to report the contribution of pharmacists to smoking cessation and study the determinants of smoking cessation success in eight pharmacies in Portugal (south) between 2009 and 2019. A real-life study was conducted with a sample of smokers who participated in pharmacist consultations. The sample included 135 smokers (average age of 47.9 ± 1.21 years), 79 (58.5%) of whom were male. In parallel with the motivation and behavioral approach, 116 (85.9%) smokers received pharmacological therapies: 108 (80.0%) were treated with nicotine replacement products and eight (5.9%) with non-nicotine medications. The interventions resulted in 70 (51.9%) smokers complying with the quit day, of whom 59 (43.7%) were smoking-abstinent at the end of the first month. Success rates were reduced to 32.6%, 28.1%, and 20.7% at the end of the 3rd, 6th, and 12th months, respectively. Smoking cessation was more successful for the participants receiving pharmacological therapies (Fisher’s exact test, *p* < 0.001) and those who participated in more pharmacist consultations (χ^2^ = 59.994, *p* < 0.001) and more telephone sessions (χ^2^ = 17.845, *p* < 0.001). Pharmacists can contribute significantly to the promotion of smoking cessation. Smokers who are more thoroughly followed up by pharmacists showed increased success rates when compared with smokers having fewer sessions with pharmacists.

## 1. Introduction

According to the Institute for Health Metrics and Evaluation, in 2017, 13,104 people in Portugal died due to causes attributable to tobacco use [1]. Nicotine dependence contributed to 19.6% of deaths from cancer, 28.1% of deaths from chronic respiratory disease, 8.7% of deaths from cerebrovascular disease and 9.8% of deaths from diabetes mellitus [1]. Despite these numbers, tobacco consumption continues to be dramatically high in Portugal, representing a significant public health problem. According to the 6th National Health Survey, in 2019, 17.0% of the Portuguese population over 15 years of age were smokers [2].

Based on several national studies, there seems to be a trend in increased motivation to quit smoking, although few people seek professional help to do so [3]. Compared to the previous year, in 2018, there was a 4.6% increase in the number of National Health System service locations providing intensive support for smoking cessation. The number of consultations and people attending these consultations increased by 11% and 13.2%, respectively. Furthermore, there was an increase in the dispensing of first-line drugs in community pharmacies to support smoking cessation [3].

However, despite such efforts, there is still a high prevalence of consumption and low cessation success rates [4]. To address this situation, it is important that health professionals in different work settings collaborate to promote smoking cessation and abstinence. Community pharmacists can contribute [5] due to their closeness to the population (from the youngest to the oldest), accessibility, and ability to regularly reinforce smoking cessation interventions [6,7].

The aim of this work is to report on pharmacists’ contribution to smoking cessation and study the determinants for smoking cessation success in the community pharmacy setting.

## 2. Materials and Methods

This is a real-life study of a sample of smokers who participated in pharmacist consultations for smoking cessation in eight community pharmacies in the south of Portugal between 2009 and 2019. The consultations were performed by an outsourced trained pharmacist, who received post-graduate training in smoking cessation. Smokers were invited to join the program during the usual activities of the pharmacy according to the following inclusion criteria:

- Being 18 years of age or older;
- Being a smoker;
- Expressed willingness to quit smoking; and
- Signed the informed consent form.

The smoking cessation program followed the recommendations of the Portuguese Directorate-General of Health [8]. These recommendations include face-to-face consultations and telephone contact (see Table 1 for a detailed description). Motivation was assessed by the Richmond test (weak (0–5); moderate (6–8); high (9,10)) [9] and dependence by the Fagerström test (low (0–3); medium (4–6); high (7–10)) [10]. Anxiety/depression signs and symptoms were assessed using the Hospital Anxiety and Depression Scale [11]. The pharmaceutical intervention focused on the motivational and behavioral approach [12] and on the prescription of pharmacological therapy (nicotine replacement products: patches, gums, lozenges, oral spray), based on the updated guidelines of the European Network for Smoking and Tobacco Prevention [13,14,15]. When necessary, smokers were referred to the physician by a written report. All participants received a type 3 medication review (according to Pharmaceutical Care Network Europe) from the pharmacist [16]. The care plan was personalized and centered on smoking cessation. However, when applicable, the pharmacist proposed interventions to solve other health problems. Cardiovascular diseases received special attention because tobacco consumption is an important risk factor. Other risk factors (e.g., dyslipidemia) were also evaluated.

Anthropometric data (weight, height, waist circumference), blood pressure, and respiratory function by simple spirometry using Spirobank II Basic™ (Medical International Research, Roma, Italy) were also evaluated and monitored.

The smoking abstinence rate was evaluated on D-Day (quit day) and at the end of the 1st, 3rd, 6th, and 12th months. Abstinence was confirmed by measuring the carbon monoxide levels in exhaled air using the GCO 100™ gas meter (Greinsinger Electronic GmbH, Regenstauf, Germany).

The applied smoking cessation program also included “follow-up after smoking cessation”. This follow-up benefitted from the fact that the patient usually continued to attend the community pharmacy for reasons other than smoking cessation consultation. Thus, each time the ex-smoker visited, it was possible to evaluate the risk of relapse and highlight the benefits of smoking cessation, promoting the maintenance of abstinence. Statistical analysis of the results was performed using SPSS statistical analysis software (IBM SPSS V25; IBM Corp, Armonk, NY, USA). For quantitative variables, measures of central tendency and dispersion measures were calculated, and for qualitative variables, relative and absolute frequencies were determined. Several bivariate analyses were performed, using the χ^2^ test and Fisher’s exact test. A type I error probability (α) of 0.05 was considered in all inferential analyses. Mean values are presented as mean ± standard error of the mean.

### Ethics Issues

Because the pharmacist in charge of patient care (M. Condinho) extracted the data from the records, and as this action has no influence on the previous smoking cessation care process, Portuguese legislation does not require the submission of this project for ethics approval. All data were anonymized for everyone else on the research team.

## 3. Results

The study was conducted on a sample of smokers participating in pharmacist consultations in eight community pharmacies over a period of ten years. The characteristics of the patients are presented in Table 2. A total of 135 patients were included in the study, 79 (58.5%) of whom were male. The participants’ ages ranged from 21 to 81 years (mean: 47.9 ± 1.21 years, median: 46.4 years). Regarding their state of health, the most prevalent comorbidities were overweight (60%), dyslipidemia (48.9%), anxiety (30.4%), and hypertension (29.9%).

Each patient participated in an average of 3.5 ± 0.28 pharmacist consultations and 2.81 ± 0.31 telephone sessions over a period of 12 months. During the consultations, more than 50% of the participants showed a moderate level of motivation to quit smoking, and their level of nicotine dependence was classified as medium (43.0%) or high (28.9%).

Regarding tobacco habits, during the first consultation, the participants reported smoking an average of 22.5 ± 0.98 cigarettes per day, with an average amount of time spent on tobacco consumption of 31.0 ± 1.24 years and an average of 1.35 ± 0.10 attempts to quit smoking. The motivational and behavioral approach was used in all pharmacist consultations. Of the 135 patients, 118 (87.4%) decided to quit tobacco with the support of pharmacological methods and 17 (12.6%) without pharmacological therapy. Nicotine replacement products were recommended in 110 cases (81.5%), and non-nicotine therapy was used by eight (5.9%) patients. Nicotine replacement products were used in oral forms by 54 patients, while 11 (10.0%) used transdermal forms, 29 used oral and transdermal forms, and 16 (14.5%) used oral forms, followed by transdermal forms.

**Figure 1:**
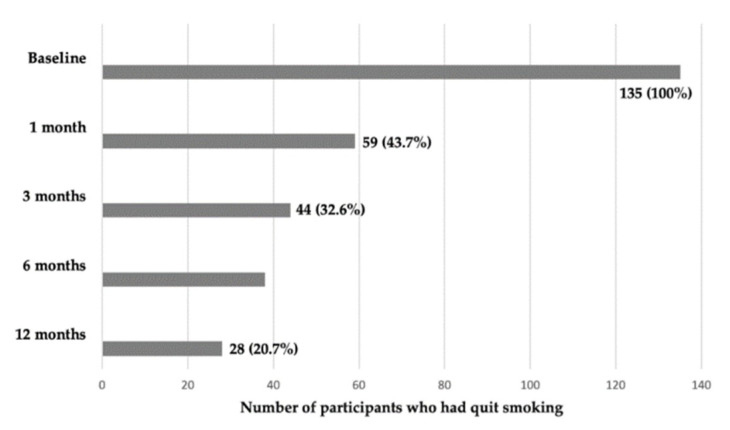
Abstinence rate (*n* = 135).

Figure 1 shows the smoking cessation success rates as a result of pharmacist intervention. Of the 135 smokers who set a day to quit tobacco use, 59 (43.7%) quit tobacco one month after. The quit smoking rate decreased to 32.6% after three months, 28.1% after six months, and 20.7% after 12 months.

The success rate measured by the number of smokers who quit tobacco was not significantly associated with sociodemographic variables such as age, gender, and education. Likewise, this rate was not affected by motivation for smoking cessation or the level of nicotine dependence (*p* > 0.05).

Smoking cessation success was significantly more frequent among patients who underwent pharmacological therapy (Fisher’s exact test, *p* < 0.001) and those who participated in more consultations (χ^2^ = 59.994, *p* < 0.001) and a greater number of telephone sessions (χ^2^ = 17.845, *p* < 0.001) (Table 3). Likewise, success was positively related to the presence of dyslipidemia (Fisher’s exact test, *p* < 0.001) and the existence of smoking habits for more than 40 years (χ^2^ = 12.403, *p* = 0.013). Conversely, the success rate was lower in patients with depression (Fisher’s exact test, *p* = 0.018). Anxious patients also had a lower success rate, although the association was not statistically significant (Fisher’s exact test, *p* = 0.060).

## 4. Discussion

Our study corroborates previously published data [5] that consider the community pharmacist a professional who can effectively contribute to the promotion of smoking cessation, increasing abstinence rates. Despite the different natures and study designs of the published interventions (motivational support only or combined with nicotine and non-nicotine drugs), our results are encouraging, with similar [17] or higher [18,19,20] abstinence rates at 6-and 12-month follow-up. However, we recognize that further research is needed with higher-quality evidence.

Regarding the factors that influence the success of smoking cessation, in our study, success was significantly more frequent among patients who underwent pharmacological therapies as well as among those who participated in more consultations and telephone sessions. Indeed, the literature correlates the success of smoking cessation programs with the frequency with which smokers complete them [21], which overlaps with greater contact with the health professional throughout follow-up (several visits or telephone sessions).

In our study, we also found that the abstinence rate was higher among patients with dyslipidemia. Other published studies have shown divergent results regarding the association between smoking cessation success rates and the presence of chronic diseases [22,23]. As dyslipidemia is an important cardiovascular risk factor and considering that the risk reduction argument was used in the context of promoting the smoking cessation program, our findings may be related to a greater awareness on the part of smokers about the risk of cardiovascular disease. However, the success rate was lower in patients with depression, which is consistent with data reported in the literature [23,24].

The relatively small sample size may be due to the smoking population’s lack of knowledge about the existence of smoking cessation programs in community pharmacies, which limits demand. It could also be explained by the insufficient awareness of pharmacy professionals regarding the potential of community pharmacists and pharmacies to promote smoking cessation. The typically weak involvement of pharmacies in tobacco control policies in Portugal should also be considered as an additional factor that could explain these data.

The biggest limitation of the present study is the absence of a control group. Controlled and randomized studies are the gold standard for evaluating the impact of any intervention. However, it is important to bear in mind that the study resulted from a service implemented in community pharmacy practice, so it is a real-life study. In fact, this study was not designed as a research study; rather, it is the result of the professional activity of the authors.

Since the selection of participants could not be carried out according to sampling rules that guarantee the representativeness of the study population, the characteristics of our sample do not totally overlap with the national reality, and thus our results cannot be generalized. Even so, they add scientific evidence about the contribution of pharmacists to the promotion of smoking cessation in the community pharmacy environment.

Regarding the characterization of the sample, the fact that the majority (58.5%) is male is consistent with the national reality, as the prevalence of smokers is higher in males than in females [2]. Conversely, the age group of the smokers was not in agreement with the national data. While at the national level the highest prevalence of smokers occurs in the 35–44 year age group (21.8%) [2], in our study, most of the participants were 40–59 years of age (44.4%). This could be because people over 40 tend to be more aware of tobacco-related diseases and more susceptible to the occurrence of chronic diseases than younger groups, for whom concerns about health problems are less significant. Thus, it is expected that the demand for smoking cessation intervention in pharmacies or requests for advice would be more common for people over the age of 40. The recruitment of participants to include in this study also justifies this higher prevalence.

Analyzing the motivation of the study participants, as would be expected by the inclusion criteria, most had moderate (53.3%) or high (20.7%) motivation, which is inconsistent with the national data, in which motivation is more evenly divided between low (41.7%) and moderate (41.3%) motivation. At the national level, only a small percentage of smokers indicate high motivation (17%) to quit smoking [25].

Regarding daily cigarette consumption, the average consumption in our sample (22.5 ± 0.98 cigarettes) was higher than that reported in the last National Health Survey [2]. This can be justified by the fact that, in our study, the average age of the smokers was close to 50 years, with an average smoking duration of more than 30 years, a high prevalence of comorbidities (including anxiety) and, therefore, possibly greater dependence. It should be noted that the average consumption recorded in our study, unlike that recorded in the smoking population at the national level, was similar to that recorded in another study carried out in Portugal with a similar sample [26], which confirms our data.

The most prevalent pathologies in our sample are in agreement with the most prevalent pathologies at the national level, although with different relative percentages [27]. The success rates reported in the literature reveal discrepant values, which, in some cases, makes our success rates more favorable than in other cases. Our success rate after one month (43.7%) compared favorably with others reported in the literature, ranging between 22% [28] and 26% [29]. After three months (32.6%), it was unfavorable when compared to another study (58%) [21]. After six months (28.1%), it was slightly higher or similar to some published studies (18%, 25%, and 28%, respectively) [17,30,31] and lower compared to another one [32]. After 12 months (20.7%), our success rate was still higher when compared to some studies [28,33], but lower compared to others (45%) [21]. This discrepancy can be explained by different study designs and sample sizes, but above all, it seems to be related to the study setting. The studies in which abstinence rates were more favorable were conducted in medical clinics, with varenicline (the most effective drug in monotherapy [34]) being the most commonly prescribed drug [21,32]. In fact, this therapeutic option is not available to pharmacists in Portugal, which justifies the use, almost entirely, of nicotine replacement therapy in our study. It is understandable that when the immediate use of varenicline is available, due to the convenience of taking it and the fact that it is already a reimbursed drug in many countries (including Portugal), the choice of this drug contributes to smoking cessation success. However, therapy with nicotine substitutes requires more attention in use and administration and is more expensive [26]. This could contribute to lower abstinence rates compared to cessation programs where varenicline is available by direct prescription.

Nevertheless, when we compared our results with those of similar smoking cessation programs in community pharmacies elsewhere [17,28,29,30,31], our abstinence rates were quite favorable and encouraging. Thus, considering the high prevalence of tobacco consumption in Portugal, the high burden of associated diseases, and the need for the involvement of all health professionals in tobacco control, our data should encourage the implementation of smoking cessation programs in community pharmacies.

## 5. Conclusions

Based on the results of this study, community pharmacists can contribute significantly to the promotion of smoking cessation. Smokers who were thoroughly followed up by pharmacists had increased success rates compared to smokers who had fewer sessions with pharmacists.

## Figures and Tables

**Table 1:** Description of face-to-face consultations and telephone sessions.

Face-to-Face Consultations and Phone Contacts	
*Action*	Description
*First appointment* *(Based on a specific formulary developed for this purpose)*	- Smoker’s identification: sociodemographic data (e.g., gender, age, education level, profession, situation regarding the profession) - Smoker’s evaluation: reason for smoking cessation, number of years of tobacco consumption, number of cigarettes consumed per day, places where he or she smokes (among other smoking habits), previous attempts, family support, fears associated with cessation, assessment of dependence, motivation, readiness for change and anxiety/depression signs and symptoms, among other relevant data - Collection of clinical and pharmacotherapeutic information: current health problems, personal and family history of illness, current medication and allergies - Lifestyle: coffee consumption, alcoholic beverages, exercise, food - Determination of anthropometric parameters, blood pressure, carbon monoxide and spirometry - Schedule D-Day - Definition of personalized therapeutic care plan
*Next appointments* *(In general, in the 2nd, 3rd and 6th months)*	- Before D-Day - Check the implementation of the agreed plan - Discuss the difficulties and define strategies to overcome them - Highlight the benefits of smoking cessation (5Rs approach) [13,14,15] - Reassess motivation and availability to initiate smoking cessation - Reassess anthropometric data and blood pressure - Schedule D-Day - After D-Day - Congratulate on success - Check for lapses - Assess nicotine withdrawal symptoms, major difficulties and adverse reactions to medication - Review lifestyles and strategies to overcome cigarette memory/cravings - Evaluate anthropometric data, blood pressure, carbon monoxide - Review the therapeutic plan; readjust if necessary
*Last appointment* *(In the 12th month)*	- Congratulate on success - Assess nicotine withdrawal symptoms - Highlight benefits and address relapse prevention strategies - Reassess anthropometric parameters, blood pressure and carbon monoxide levels
*Telephone contact*	- D-Day - Confirm smoking cessation and congratulate - Review adherence to the defined therapeutic care plan - Evaluate potential adverse reactions - Encourage abstinence - After D-Day - Confirm abstinence and congratulate - Evaluate difficulties in the process - Evaluate pharmacological therapy, if still present - Encourage the smoker to maintain abstinence, emphasizing the benefits

**Table 2:** Characteristics of the enrolled patients.

	Frequency	Percentage
Age group		
21–39	37	27.4
40–59	60	44.4
≥60	38	28.1
Gender		
Male	79	58.5
Female	56	41.5
Education		
<Middle school	2	1.5
Middle school	59	43.7
Secondary	49	36.3
Higher	23	17
NA	2	1.5
Work situation		
Active	100	74.1
Retired	25	18.5
Unemployed	6	4.4
Student	3	2.2
NA	1	0.7
Motivation for smoking cessation		
Weak	25	18.5
Moderate	72	53.3
High	28	20.7
NA	10	7.4
Age of tobacco use initiation		
9–11	20	14.8
12–15	39	28.9
16–20	58	43
>20	18	13.3
Time spent on tobacco use (years)		
7–10	9	6.7
11–20	27	20
21–30	33	24.4
31–40	30	22.2
>40	36	26.7
Nicotine dependence		
Low	29	21.5
Medium	58	43
High	39	28.9
NA	9	6.7

Note: NA—missing observations.

**Table 3:** Factors influencing smoking cessation success.

	Abstinent N (%)	Non-Abstinent N (%)	Total N (%)	Statistical Significance
Age group (years)				
21–39	20 (54.1)	17(45.9)	37 (100)	χ^2^ = 8.772,
40–59	26 (44.1)	33 (55.9)	59 (100)	*p* = 0.119
≥60	24 (63.2)	14 (2)	38 (100)	
Gender				Fisher’s exact test,
Male	33 (41.8)	46 (58.2)	79 (100)	*p* = 0.084
Female	32 (57.1)	24 (42.9)	56 (100)	
Education				
<Middle + middle school	27 (44.3)	34 (55.7)	61 (100)	
Secondary	27 (56.3)	21 (43.8)	48 (100)	χ^2^ = 4.607
Higher	16 (69.6)	7 (30.4)	23 (100)	*p* = 0.099
Motivation for cessation				
Weak	12 (48)	13 (52)	25 (100)	
Moderate	36 (50.7)	35 (49.3)	61 (100)	χ^2^ = 1.052,
High	17 (60.7)	11 (39.3)	28 (100)	*p* = 0.588
Nicotine dependence				
Low	16 (55.2)	13 (44.8)	29 (100)	
Medium	27 (47.4)	30 (52.6)	57 (100)	χ^2^ = 0.911,
High	22 (56.4)	17 (43.6)	39 (100)	*p* = 0.661
Pharmacological therapy				Fisher’s exact test,
Yes	68 (58.6)	48 (41.4)	116 (100)	*p* < 0.001
No	2 (11.1)	16 (88.9)	18 (100)	
No. of consultations				
1	4 (9.1)	40 (90.9)	44 (100)	χ^2^ = 59.994,
2–3	23 (54.8)	19 (45.2)	42 (100)	*p* < 0.001
4–5	18 (85.7)	3 (14.3)	21 (100)	
More than 5	25 (92.6)	2 (7.4)	27 (100)	
No. of telephone sessions				
None	3 (16.7)	15 (83.3)	18 (100)	
1–2	29 (47.5)	32 (52.5)	61 (100)	χ^2^ = 17.845,
3–4	21 (61.8)	13 (38.2)	34 (100)	*p* < 0.001
More than 4	17 (81.0)	4 (19.0)	21 (100)	
Time spent using tobacco (years)				
7–10	6 (66.7)	3 (33.3)	9 (100)	
11–20	12 (44.4)	15 (55.6)	27 (100)	χ^2^ = 12.403,
21–30	17 (51.5)	16 (48.5)	33 (100)	*p* = 0.013
31–40	9 (31.0)	20 (69.0)	−100	
More than 40	26 (72.2)	10 (27.8)	−100	
Dyslipidemia				Fisher’s exact test,
Yes	44 (67.7)	21 (32.3)	65 (100)	*p* < 0.001
No	26 (37.7)	43 (62.3)	69 (100)	
Anxiety				Fisher’s exact test,
Yes	16 (39.0)	25 (61.0)	41 (100)	*p* = 0.060
No	54 (58.1)	39 (41.9)	93 (100)	
Depression				Fisher’s exact test,
Yes	6 (27.3)	16 (72.7%)	22 (100)	*p* = 0.018
No	64 (57.1)	48 (42.9%)	112 (100)	

## Data Availability

Not applicable.
